# Duration of Wound Coverage for the Prevention of Surgical Site Infections After Surgery: A Systematic Review of Current Evidence With Meta‐Analysis of Randomised Controlled Trials

**DOI:** 10.1111/iwj.70943

**Published:** 2026-05-12

**Authors:** Moira D. Cruickshank, Kirsty Loudon, Paul D. Manson, George Ramsay, Miriam G. Brazzelli

**Affiliations:** ^1^ Aberdeen Centre for Evaluation, Institute of Applied Health Sciences University of Aberdeen Aberdeen UK; ^2^ Freelance Researcher Edinburgh UK; ^3^ Department of Surgery Aberdeen Royal Infirmary Aberdeen UK

**Keywords:** dressings, surgical site infection, systematic review

## Abstract

This systematic review aimed to identify the published evidence on the length of time surgical wounds should be covered with dressings after surgery to prevent surgical site infections (SSI). MEDLINE, Embase, CINAHL and CENTRAL were searched in August 2025 for RCTs comparing the effects of different lengths of time wounds are covered after surgery on SSI at specified time points. Sixteen RCTs met the inclusion criteria. Duration of dressing and patient population varied across studies. The SSI rate was higher in groups with longer dressing duration in nine studies, higher in the shorter duration group in three studies and equivalent between groups in one study. Two studies reported no SSIs, and one study reported a composite outcome. Meta‐analysis of results showed no significant difference in the development of SSI between the shorter and longer dressing duration groups (RR 0.84, 95% CI: 0.57, 1.22, *p* = 0.33). The GRADE assessment of evidence was very low. Studies were heterogeneous in terms of patient population, type of surgery and dressing duration. Our findings add to the evidence showing that shorter and longer dressing durations are comparable in terms of SSI risk. Future research should prioritise well‐powered RCTs examining standardised dressing durations and follow‐up, with stratification by wound characteristics and dressing material.

## Key Points

- Surgical site infections (SSI) can be devastating for patients and health services alike and prevention is a key aim of contemporary surgical practice.
- Wound dressings constitute one approach to prevention but there are currently no definitive guidelines for timing of dressing removals.
- Sixteen randomised controlled trials comparing different durations of wound dressings across varied patient populations showed that shorter and longer dressing durations are comparable in terms of SSI risk.
- Well‐powered trials are needed to examine standardised dressing durations and follow‐up.

## 1 Introduction

The rate of surgical site infections (SSI) varies by type of surgery, contamination status and patient co‐morbidity. However, once they have occurred, SSI can have far‐reaching consequences for patients and health services alike. SSI prevention is thus a key aim of any contemporary surgical practice. Current multi modal evidence‐based approaches may prevent around half of all SSI, with wound dressings being part of these bundles of measures [1, 2, 3]. However, one under assessed question is for how long a dressing should remain on a surgical wound.

Whilst guidelines on preventing SSI generally conclude that there is no robust evidence for the timing of dressing removal, several recommend keeping dressings in place for 48 h [4, 5, 6, 7, 8]. There are advantages and disadvantages to early removal of dressings (within 48 h after surgery) and delayed removal (at least 48 h after surgery). For example, the wound healing process can be assisted by the moist conditions created by a dressing, but a moist environment combined with excessive wound exudate can lead to maceration of the wound and delayed healing [1, 9, 10]. Thus, there is a balance for clinicians when deciding on an appropriate dressing between protecting a wound/promoting healing and avoiding issues such as maceration.

The available literature on the optimal timing for wound dressing removal after surgery to prevent surgical site infections remains unclear. Two systematic reviews comparing early dressing removal (within 48 h post‐surgery) with delayed removal (after 48 h) concluded that there was no significant difference between early and delayed dressing removal regarding SSI, wound dehiscence, adverse events or patient satisfaction [1, 2]. Despite this, neither review specifically addressed how long wounds should remain covered post‐surgery. Therefore, this systematic review is necessary to explore the literature on the optimal duration for wound coverage with dressings to prevent SSI. This systematic review aims to address the following research questions:

1. *What is the current evidence regarding the optimal duration of postoperative wound dressing to prevent SSI?*
2. *To what extent is the duration of postoperative wound coverage influenced by type of dressing, wound characteristics, type of surgery or a combination of these factors?*

## 2 Methods

An objective synthesis of the evidence was conducted according to current methodological standards [11] and reported in line with the PRISMA 2020 checklist [12]. The methods were pre‐specified in a research protocol published in the Research Registry (http://bit.ly/45QY8Rg; Unique Identifying Number reviewregistry1906).

### 2.1 Eligibility Criteria

This review included published randomised controlled trials (RCTs) conducted in any relevant clinical setting and comparing the effects of different lengths of time wounds are covered after surgery (with the same type of dressing in each group) on outcomes at specified time points. Patients with a wound resulting from a surgical incision were eligible for inclusion. Specifically, the following were eligible for inclusion: RCTs comparing different durations of the same dressing (where randomisation is based on the duration of wound dressing); RCTs comparing dressing of a specified duration with no dressing; RCTs comparing glue plus dressing to glue‐only; RCTs involving minimally invasive surgery, including laparoscopic procedures and minor skin excisions, with a focus on the duration of dressing.

Research focusing on in vitro studies, chronic wounds or wound healing by secondary intention was excluded. Additionally, studies involving wounds left open during the operation and requiring prolonged hospitalisation, epidermal skin grafts, endoscopic sinus surgery or military wounds were outside the scope of this systematic review. Studies examining the frequency of dressing changes, immediate revision surgery (within 1 month of the original procedure), arthrodesis or specific niche dressings (e.g., Robert Jones bandages) did not align with the objectives of this systematic review and were not included. Specialised procedures (e.g., anal fistulas, diabetic foot procedures, pilonidal sinus) and skin graft donor sites were also excluded as these often involve unique wound management considerations. Lastly, studies that incidentally report dressing duration as part of comparisons between different dressings were excluded to maintain a focus on research explicitly addressing dressing duration.

Outcomes of interest were surgical site infection (SSI) (including up to 30 days and 1 year), wound opening, unintended readmission to hospital, reoperation for wound dehiscence, length of hospital stay, length of time for wound healing, quality of life and mortality. In addition, aspects considered relevant were type or size of wounds, anatomical location of the wound, type of surgery (elective versus emergency; clean/clean contaminated/contaminated/dirty), type of dressing and whether antibiotics have been administered or not (both prophylactic and as treatment for resulting SSI).

### 2.2 Information Sources

An Information Specialist developed a comprehensive search strategy including database index terms and free‐text keywords covering surgical wounds, dressings and timing to identify relevant published studies. The last searches were performed on 28th August 2025. The databases searched were MEDLINE, Embase, the Cumulative Index to Nursing and Allied Health Literature (CINAHL) and the Cochrane Library, including the Database of Systematic Reviews and the Central Register of Controlled Trials (CENTRAL). No restrictions were placed on study type or language during the search phase, but the search was limited to studies published from 1989 onward. As the literature search yielded a large number of citations, we divided them by publication year: 1989–2000 and 2000 onwards. We then focused on the 2000 onwards results, as they provided sufficient evidence for this assessment and the resources needed for evaluating the remaining literature were prohibitive. Additionally, clinical trial registries, including ClinicalTrials.gov and the WHO International Clinical Trials Registry Platform, were searched for ongoing studies. Reference lists of included studies and relevant systematic reviews were checked for eligible studies. The searches are given in Appendix 1.

### 2.3 Selection Process

Following pilot testing, one reviewer (MC) screened all citations identified by the search strategies for eligibility. A second reviewer (MB) independently checked a random 10% sample for eligibility. Potentially relevant articles were retrieved in full and were screened by one reviewer (MC) with an independent 20% random check by a second reviewer (KL). Reasons for the exclusion of full‐text articles were recorded. At all stages of the selection process, disagreements were resolved through discussion.

### 2.4 Data Collection Process

A data extraction form was designed specifically for this assessment. Following piloting, data from included studies were independently extracted by one reviewer (MC or KL). A second reviewer (MC or KL) crossed‐checked the extracted data to ensure accuracy. Any disagreements were resolved through discussion to reach a consensus. Data were collected on the following: study characteristics (country, setting, inclusion/exclusion criteria, number of participants randomised and analysed, number and reasons for dropouts); participant characteristics (age, sex, BMI, type of surgery and antibiotics administered); intervention and comparator details. Where available, data were extracted for the following outcome measures: surgical site infection within (including up to 30 days and 1 year); wound dehiscence; unintended readmission to hospital; reoperation for wound dehiscence; length of hospital stay; length of time for wound healing; quality of life; mortality.

### 2.5 Risk of Bias Assessment

Two reviewers independently assessed the risk of bias of included studies for the outcome SSI using the revised Cochrane Risk of Bias tool for randomised trials (RoB2) [13]. Disagreements were resolved through discussion or arbitration.

### 2.6 Certainty of Evidence Assessment

Certainty of evidence for the outcome SSI was assessed using the GRADE approach using GRADEpro GDT software. All included RCTs were initially assessed as high‐certainty evidence and downgraded based on risk of bias, inconsistency, indirectness, imprecision and publication bias. Two reviewers jointly conducted the GRADE assessment.

### 2.7 Synthesis Methods

The findings of each included study were tabulated and summarised narratively for each outcome of interest. For the purpose of this review, intervention and comparator treatments were classified such that the comparator represented the longer dressing duration while the intervention was defined as the shorter dressing duration or no dressing. Where sufficient data were available, and pooling was considered appropriate, random‐effects meta‐analyses were conducted using the inverse variance method. Statistical heterogeneity between studies was assessed using the *I* ^2^ statistic and between‐study variance was estimated using the Restricted Maximum Likelihood (REML) method.

## 3 Results

### 3.1 Study Selection

**FIGURE 1:**
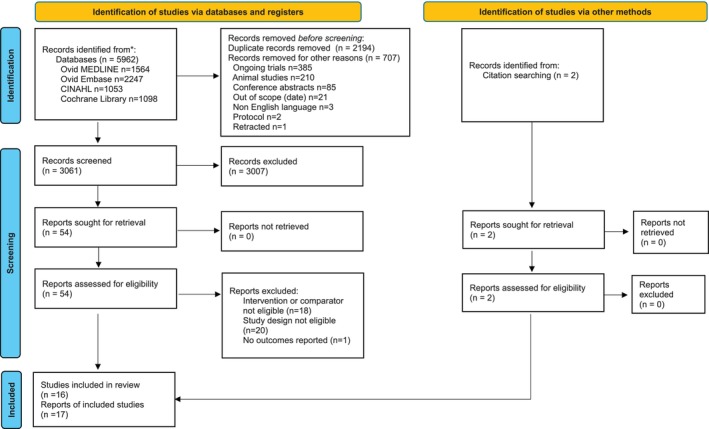
PRISMA flow diagram. *Source:* Page et al. [12]. For more information, visit: http://www.prisma‐statement.org/ .

The database searches identified 3061 unique publications and two further publications were identified from the reference list of a systematic review. A total of 54 publications were selected for full‐text screening, of which 17, reporting a total of 16 RCTs, met our inclusion criteria. A PRISMA flow diagram detailing the study selection process is presented in Figure 1.

Separately, the Cochrane CENTRAL database was searched for ongoing trials. A total of 342 trials were found from ClinicalTrials.gov and the World Health Organisation International Clinical Trials Registry Platform (ICTRP). Three relevant ongoing trials were identified.

### 3.2 Characteristics of Included Studies

A total of 16 studies (all RCTs) reported across 17 publications were included in this systematic review. The characteristics of the included studies are detailed in Appendix 2.

Seven studies reported clean surgery, including: breast augmentation; [14] breast reconstruction; [15] breast reduction; [16] facial melanocytic excision; [17] carpal tunnel release; [18] pinnaplasty (ear reshaping surgery); [19] minor skin excision [20].

Nine studies reported clean‐contaminated surgeries: Caesarean section; [21, 22, 23, 24, 25, 26, 27, 28] thoracic surgery [29].

Eight studies used gauze dressings [14, 15, 16, 21, 26, 27, 28, 29]. Other dressing types included: vaselinated gauze [17]; medicated gauze [18]; melolin (a type of low adherent absorbent dressing [20]); standard head bandages [19]; standard non‐woven dressings [24]; compressive dressings [25].

One study used absorbent pad for the comparator dressing and an intervention involving no dressing, leaving the wound exposed wherever possible [22] One study did not report details of the type of dressing used [23].

### 3.3 Characteristics of Participants

Baseline characteristics of participants are reported in Appendix 3. Most studies included exclusively female participants due to the nature of the surgical procedures [14, 15, 16, 21, 22, 23, 24, 25, 26, 27, 28]. In two studies, the majority of participants were female [17, 18]. The remaining three studies included similar proportions of male and female participants [19, 20, 29].

In studies involving caesarean section, the mean age of participants ranged from 28.1 years [26] to 32.9 years [24]. Among the three studies that focused on breast surgery, the mean age of participants ranged from 27.9 years [14] to 49.3 years [15]. One pinnaplasty study exclusively recruited participants younger than 16 years, reporting mean ages of 10 years in the intervention group and 9 years in the comparator group [19]. In the remaining studies, the mean age ranged from 28 years [17] to 56.5 years [20]. Prophylactic antibiotics were administered in nine studies [14, 15, 21, 22, 24, 25, 26, 28, 29]. Antibiotics were not administered in three studies [16, 17, 20] and this information was not reported in the remaining four studies [18, 19, 23, 27]. Only one study reported details of the ethnicity of participants, with around two‐thirds being described as Malay in both groups [22].

### 3.4 Risk of Bias Assessments

**FIGURE 2:**
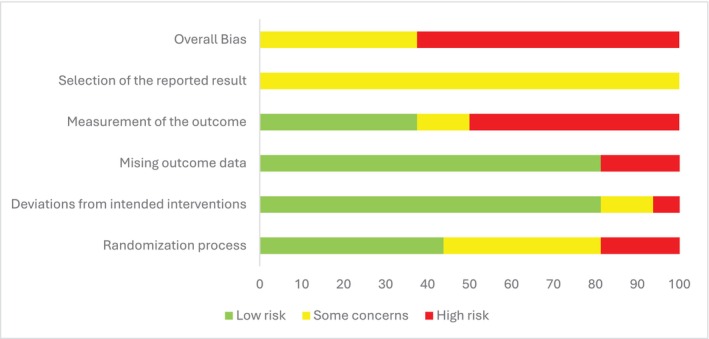
Summary of risk of bias assessments.

Figure 2 presents a summary of the risk of bias assessments of the 16 included studies for the outcome SSI.

Risk of bias assessments of individual studies are presented in the Supporting Information. According to the Cochrane risk of bias tool (version 2), overall risk of bias was high for 10 trials [18, 21, 22, 23, 24, 25, 26, 27, 28, 29] and there were some concerns for the remaining six trials [14, 15, 16, 17, 19, 20].

### 3.5 Certainty of Evidence Assessment

The Summary of Findings table for the main outcome of interest (SSI) assessed using the GRADE approach is presented as Table 1.

**TABLE 1:** Summary of findings table.

Outcome	Anticipated absolute effects		Relative effect (95% CI)	No of participants (Studies)	Certainty of evidence
	Risk with early removal	Risk with delayed removal			
SSI	83 per 1000 (57–121)	99 per 1000	0.84 (0.57–1.22)	5590 (16 RCTs)	⊕◯◯◯ Very low^a^, ^b^, ^c^, ^d^

^a^ 10/16 studies were at high risk of bias overall and 8/18 at high risk of bias for measurement of the outcome.

^b^ Statistically significant heterogeneity in overall effect estimate.

^c^ Included studies answer research question.

^d^ Wide CIs in included studies.

### 3.6 Clinical Outcomes

#### 3.6.1 Surgical Site Infection (SSI)

All 16 included studies reported SSI as an outcome (Table 2). The relative proportions of participants experiencing SSI in the intervention (shorter duration of dressing) and comparator groups (longer duration of dressing) varied across studies. The SSI rate was higher in the comparator group in nine studies [17, 19, 20, 22, 23, 25, 26, 27, 29] higher in the intervention group of three studies [15, 16, 24] and equivalent between the groups in one study [28] No SSI was reported in either intervention or comparator group in two studies [14, 18] One study reported similar proportions of participants with wound score > 0 at 1 week but more participants in the intervention group with such a score at 6 weeks [21]

**TABLE 2:** Surgical site infection rate in the 16 included studies.

Study ID	SSI intervention group, n/N (%)	SSI comparator group, n/N (%)	SSI definition	Timepoint of SSI assessment
Clean surgery				
Mendes et al. [14]	(1 day) 0/40 (0)	(6 days) 0/38 (0)	CDC criteria	1 year
Veiga et al. [15]	(1 day) 6/94 (6.4)	(6 days) 3/92 (3.3)	CDC criteria	30 days
Ghandi et al. [17]	(24 h) 6/234 nevi (2.6)	(48 h) 8/234 nevi (3.4)	NR	4–6 days
Ritting et al. [18]	(48–72 h) 0/45 (0)	(2 weeks) 0/49 (0)	NR	NR
Veiga‐Filho et al. [16]	(1 day) 7/35 (20.0)	(6 days) 2/35 (5.7)	CDC criteria	30 days
Heal et al. [20]	(12 h) 8.4% (37/442 back calculated)^a^	(48 h) 8.9% (37/415 back calculated)^a^	Infection must be within 30 days of excision; There must be purulent discharge from the wound or the general practitioner must diagnose a wound infection	Day of removal of sutures (or sooner if patient re‐presented with a perceived infection)
Ramkumar et al. [19]	(24 h) 0/39 (0)	(10 days) 1/39 (2.6)	NR	10 days
Clean‐contaminated surgery				
Khlifi et al. [25]	(2 days) 7/200 (3.5)	(10 days) 20/200 (10.0)	CDC and NHSN criteria	30 days
Kilic et al. [21]	(24 h) Wound score > 0: discharge 136/430 (31.6); 1 week 141/430 (32.8); 6 weeks 38/430 (8.8)	(48 h) Wound score > 0: discharge 143/439 (32.6); 1 week 123/439 (28.0); 6 weeks 17/439 (3.9)	Modified 1 day ASEPSIS score	Discharge, 1 week, 6 weeks
Wadhwa et al. [26]	(4 days) 40/250 (16)	(8 days) 80/250 (32)	NR	1 month
El‐Sayed et al. [27]	(6–12 h) 9/64 (14.1)	(5 days) 25/64 (39.1)	Presence of purulent drainage, pain or tenderness, localised swelling, erythema or fever	2 weeks
Tan et al. [22]	(No dressing) Superficial SSI up to 28 days 2/153 (1.3); Superficial SSI at discharge 0/165 (0); Superficial SSI on day 14 2/165 (1.2); Superficial SSI on day 28 0/153 (0) Deep incisional SSI: zero Space/intra‐organ infections: zero	(1 day) Superficial SSI up to 28 days 5/157 (3.2); Superficial SSI at discharge 0/166 (0); Superficial SSI on day 14 5/166 (3.0); Superficial SSI on day 28 0/157 (0) Deep incisional SSI: zero Space/intra‐organ infections: zero	CDC criteria	14 and 28 days (for superficial SSI) NR for deep incisional and space/intra‐organ infections
Nesrallah et al. [23]	(12–30 h) 21/300 (7.0)	(30–48 h) 29/302 (9.6)	Surgical site disruption or infection	6 weeks
Chandrasiri and Fernandopullae [28]	(6–12 h) 19/119 (16.0)	(24–36 h) 45/281 (16.0)	CDC/NHSN criteria	2 weeks
Peleg et al. [24]	(6 h) 8/160 (5.0)	(24 h) 6/160 (3.8)	Wound infection was diagnosed if there was purulent discharge	5–7 days
Zhou et al. [29]	(48 h) Superficial: 6/115 (5.2) Deep: 0/115 (0)	(7–8 days) Superficial: 7/115 (6.1) Deep: 0/115 (0)	Wound infection was defined as either superficial or deep, in conjunction with one of the following: excision of wound tissue, a positive wound culture finding or treatment with antibiotics	30 days

*Note:* No PRISMA flowchart available (Figure 2 is a duplicate of Figure 1) and numbers randomised reported in the abstract appear to be the wrong way round. Four hundred and fifty were randomised to WET intervention and 420 to DRY comparator. Thirteen were lost to follow‐up, believed to be eight from WET intervention and five from DRY intervention, leaving 442 and 415 respectively.

Abbreviation: NHSN, National Healthcare Safety Network.

^a^ Percentages reported for SSI and denominators were unclear due to inconsistent reporting.

#### 3.6.2 Meta‐Analysis: SSI

**FIGURE 3:**
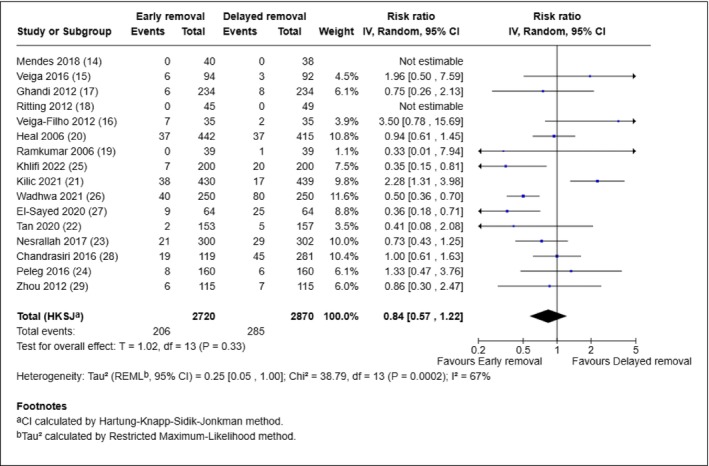
Meta‐analysis for incidence of SSI.

All 16 included studies reported the rate of SSI as an outcome and were combined in a meta‐analysis. The study by Kilic 2021 reported a composite outcome using the modified ASEPSIS score at discharge, 1 week and 6 weeks [21]. For the meta‐analysis of SSI, wound score > 0 at 6 weeks was chosen as this time point most closely aligns with the standard 30‐day definition of SSI. Figure 3 presents the forest plot with the studies ordered as follows: first, the seven ‘clean surgery’ trials [14, 15, 16, 17, 18, 19, 20] followed by the nine ‘clean‐contaminated surgery’ trials [21, 22, 23, 24, 25, 26, 27, 28, 29] Within these surgery categories, studies are further grouped by organ type: breast [14, 15, 16] skin [17, 20] carpal tunnel [18] ears [19] caesarean section [21, 22, 23, 25, 26, 27, 28] and thoracic [29].

Overall, there was no significant difference in the proportion of participants developing SSI between the early removal and delayed removal groups, with a trend towards lower risk for the early removal (intervention; RR 0.84, 95% CI: 0.57, 1.22, *p* = 0.33) but certainty of evidence was rated as very low and there was evidence of statistically significant heterogeneity (*I* ^2^ = 67%, *p* = 0.0002) in the analysis. In particular, the certainty of evidence for SSI was downgraded due to 10/16 studies being at high risk of bias overall, statistically significant heterogeneity in the effect estimate and wide confidence intervals in the included studies. There was statistically significant heterogeneity (*I* ^2^ = 67%, *p* = 0.0002). Among the two breast surgery trials that reported SSI events, fewer infections were observed in the delayed removal group [15, 16]. In contrast, the two skin‐related trials showed a broadly similar proportion of SSI between intervention groups with a slightly higher number of infections reported in the delayed removal group [17, 20]. Of the eight trials that focused on caesarean section surgery [21, 22, 23, 24, 25, 26, 27, 28] five favoured early removal [22, 23, 25, 26, 27] two favoured delayed removal [21, 24] and the remaining trial showed equivalent proportions of SSI between groups [28].

A sensitivity analysis was conducted, removing the study by Kilic, [21] because it reported a composite outcome rather than a direct measure of SSI, and included multiple timepoints that did not align with the standard 30‐days definition of SSI. Excluding this study resulted in an effect estimate consistent with that of the primary analysis (RR 0.73, 95% CI: 0.52, 1.02, *p* = 0.06), with moderate heterogeneity (*I* ^2^ = 46%, *p* = 0.03).

A further sensitivity analysis was conducted, excluding trials assessed as having a high overall risk of bias [18, 21, 22, 23, 24, 25, 26, 27, 28, 29] The effect estimate remained non‐significant (RR 1.03, 95% CI: 0.59 to 1.79, *p* = 0.89).

We also combined the results of the three studies involving breast surgery for the outcome SSI. All three were conducted in Brazil and involved ‘clean’ surgery of the breast: breast augmentation [14] breast reconstruction [15] and breast reduction [16]. The study involving breast augmentation [14] assessed SSI at 12 months, and the reconstruction and reduction studies at 30 days [15, 16]

**FIGURE 4:**
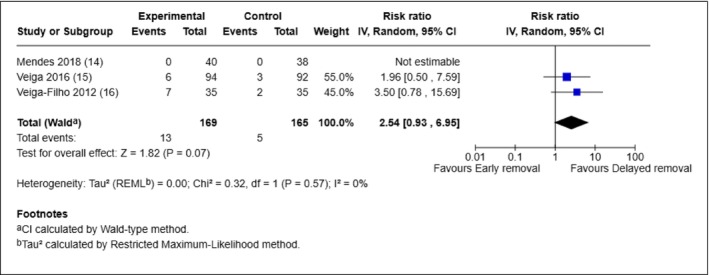
Meta‐analysis of SSI rate in three breast surgery trials.

Figure 4 shows that participants in the early removal group were more likely to experience SSI than those in the delayed group, but there is no evidence of a statistically significant difference between groups (RR 2.54, 95% CI: 0.93 – 6.95, *p* = 0.07). No statistical heterogeneity was observed among studies (*I* ^2^ = 0%, *p* = 0.57). In one study, no infections were observed in either group [14].

**FIGURE 5:**
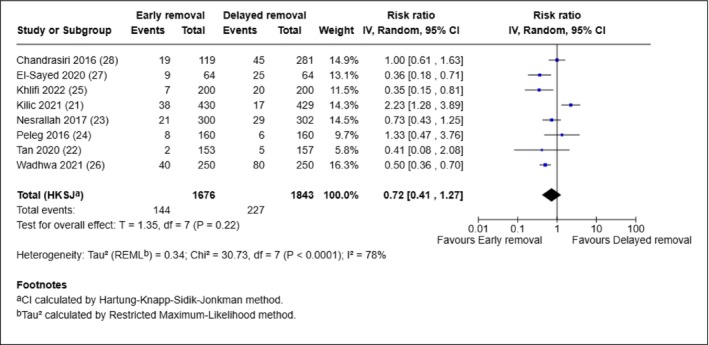
Meta‐analysis of SSI in eight caesarean section trials.

Results of the eight studies involving caesarean section were combined in a meta‐analysis (Figure 5). Participants in the early removal group were less likely to experience SSI than those in the delayed removal group. However, the difference was not statistically significant (RR 0.72, 95% CI: 0.41, 1.27, *p* = 0.22). Statistical heterogeneity was substantial (*I* ^2^ = 78%, *p* < 0.0001). It is worth noting that the duration of dressing varied across the eight studies.

### 3.7 Clinical Outcomes

Table 3 presents details of the clinical outcomes reported across the included studies. Two outcomes specified in the protocol—reoperation for wound dehiscence and mortality—were not reported by any of the included studies.

**TABLE 3:** Clinical outcomes reported across included studies.

Study ID	Intervention group	Comparator group	Outcome definition/notes
Wound dehiscence, n/N (%)			
Clean surgery			
Ghandi et al. [17]	2/106 (1.9)	0/105 (0)	Open wound
Ritting et al. [18]	0/45 (0)	1/49 (2.0)	Slight wound dehiscence
Ramkumar et al. [19]	2/39 (5.1)	1/39 (2.6)	Minor dehiscence not requiring intervention
Clean‐contaminated surgery			
Chandrasiri and Fernandopullae [28]	4/205 (2.0)	3/195 (1.5)	Wound dehiscence
Peleg et al. [24]	8/160 (5.0)	5/160 (3.1)	Dehiscence or disruption if there was clear separation of superficial or deep layers, OR (95% CI) 1.63 (0.52, 5.10)
Zhou et al. [29]	2/115 (1.7)	1/115 (0.9)	Mild wound separation, *p* = 0.561
Unintended readmission to hospital, n/N (%)			
Clean surgery			
Veiga et al. [15]	4/94 (4.3)	1/92 (1.1)	Five patients with deep incisional SSI who had undergone implant reconstruction and required readmission to remove the implant
Ramkumar et al. [19]	8/NR (Slipped bandage: 3, haematoma: 1, oozing/soaking: 1, agitated/ unhappy: 4)	14/NR (Slipped bandage: 10, oozing/soaking: 3, itching: 1, agitated/unhappy: 1)	Reported as ‘unscheduled visits’. Denominators unclear.
Clean‐contaminated surgery			
Tan et al. [22]	0/153 (0)	1/157 ( < 1)	Readmission for wound opening
Peleg et al. [24]	3/160 (1.9)	3 (1.9)	Women were readmitted if intravenous antibiotics were deemed necessary OR (95% CI) 1.00 (0.20–5.03)
Length of hospital stay, days, mean (SD)			
Clean‐contaminated surgery			
Khlifi et al. [25]	50.1 (8.3) h	52.6 (24.3) h	Duration of postoperative hospitalisation, *p* = 0.173
Kilic et al. [21]	2 (0.4)	2.1 (0.5)	Length of hospital stay
Wadhwa et al. [26]	5.7 (2.1)	10.1 (2.7)	Hospital stay, *p* < 0.0001
El‐Sayed et al. [27]	1 day: 90.6% > 1 day: 9.4%	1 day: 57.8% > 1 day: 42.2%	Hospital stay, *p* < 0.001
Peleg et al. [24]	Median (range) 4 (3–9)	Median (range) 4 (3–12)	Duration of hospital stay, *p* = 0.65
Zhou et al. [29]	8.3 (1.1)	8.4 (3.7)	Postoperative length of stay, *p* = 0.760
Length of time for wound healing, days, *n* (%)			
Clean surgery			
Ghandi et al. [17]	81 (76.4)/19 (17.9)/4 (3.8)	75 (71.4)/23 (21.9)/7 (6.7)	Close wound/mature scar/scab or scar
Clean‐contaminated surgery			
Wadhwa et al. [26]	6.6 (1.9)	10.6 (2.1)	Duration of healing, *p* < 0.0001
Zhou et al. [29]	106/115 (92.2)	107/115 (93.0)	Normal surrounding skin integrity by day 30, *p* = 0.801

*Wound dehiscence* was reported by six studies [17, 18, 19, 24, 28, 29] but the definition of wound dehiscence varied across studies. Rates of wound dehiscence were generally low but tended to be higher in the intervention groups [17, 19, 24, 28, 29] except for one study [18].

*Unintended readmission to hospital* was reported by four studies with differing reasons [15, 19, 22, 24]. One study referred to ‘unscheduled visits’, which appear to involve minor dressing‐related issues and it was unclear if these visits constituted formal readmission [19]. For the other three studies, reasons for readmission included removal of the implant in breast reconstruction patients, [15] wound opening [22] or a need for intravenous antibiotics [24].

*Length of hospital stay* was reported by six studies, with no differences observed between the intervention and comparator groups in four of them [21, 24, 25, 29] and statistically significantly shorter lengths of stay in the early removal group as compared to the delayed removal group in the remaining two studies [26, 27].

*Length of time for wound healing* was reported in three studies. One study assessed this based on closed wound, mature scar or presence of scab/scar, [17] while another defined it as restoration of normal surrounding skin integrity by Day 30 [29]. In both these studies, the proportions of participants achieving these outcomes were similar between groups. The third study reported significantly shorter duration of healing for the early removal group than the delayed removal group, albeit with no definition of the outcome reported [26].

### 3.8 Patient‐Reported Outcomes

Table 4 presents the patient‐reported outcomes reported across the included studies. Six studies reported quality of life using investigator‐generated items that focused on perceptions of safety, comfort or convenience of dressing, wear time [15, 16] or overall patient satisfaction [19, 22, 24, 25] In the breast reconstruction and breast reduction surgery studies, patients preferred having a longer dressing wear time (6 days), which was perceived to be safer [15, 16]. In the study involving correction of prominent ears and one caesarean section study, no differences in satisfaction were reported between groups [19, 22]. However, in four other caesarean section studies, greater proportions of women in the early removal groups reported higher levels of satisfaction or comfort compared to the delayed removal groups [24, 25, 27, 28].

**TABLE 4:** Patient‐reported outcomes reported across included studies.

Study ID	Intervention group	Comparator group	Outcome definition/notes
Quality of life, *n* (%)			
Clean surgery			
Veiga et al. [15]	42/82 (51.2)/23/82 (28.0)/17/82 (20.7)	64/81 (79.0)/13/81 (16.0)/4/81 (4.9)	Perceptions of safety related to dressing wear time (excellent/very good/good), *p* < 0.05
	43/82 (52.4)/24/82 (29.2)/15/82 (18.3)	46/81 (56.8)/20/81 (24.7)/15/81 (18.5)	Perceptions of comfort related to dressing wear time (excellent/very good/good), *p* > 0.05
	59/82 (72.0)/20/82 (24.4)/3/82 (3.7)	46/81 (56.8)/20/81 (24.7)/15/81 (18.5)	Perceptions of convenience related to dressing wear time (excellent/very good/good), *p* > 0.05
	68/82 (82.9)/14/82 (17.1)	7/81 (8.6)/74/81 (91.4)	Patient preference for dressing wear time (1 day/6 days), Chi^2^ test comparing groups *p* < 0.0001
Veiga‐Filho et al. [16]	15/35 (42.9)/14/35 (40.0)/6/35 (17.1)/0/35 (0)	30/35 (85.7)/4/35 (11.4)1/35 (2.9)/0/35 (0)	Perceptions of safety related to dressing wear time (excellent/very good/good/fair), *p* < 0.05
	16/35 (45.7)/8/35 (22.9)/9/35 (25.7)/2/35 (5.7)	15/35 (42.9)/12/35 (34.3)/1/35 (2.9)/7/35 (20.0)	Perceptions of comfort related to dressing wear time (excellent/very good/good/fair), *p* > 0.05
	15/35 (42.9)/10/35 (28.6)/9/35 (25.7)/1/35 (2.9)	17/35 (48.6)/11/35 (31.4)/3/35 (8.6)/4/35 (11.4)	Perceptions of convenience related to dressing wear time (excellent/very good/good/fair), *p* > 0.05
	23/35 (65.7)/12/35 (34.3)	29/35 (82.9)/6/35 (17.1)	Patient preference for dressing wear time (1 day/6 days) Chi^2^ test comparing groups, *p* = 0.000
Ramkumar et al. [19]	36/39 (92.3)/2/39 (5.1)/1/39 (2.6)/0 (0)	34/39 (87.2)/4/39 (10.3)/0/30 (0)/1/39 (2.6)	Patient satisfaction at 10 days (happy/OK/unsatisfactory/missing) Proportion responding ‘happy’ vs. all other responses, *p* = 0.49
	32/39 (82.1)/0/39 (0)/ 0/39 (0)/7/39 (17.9)	31/39 (79.5)/3/39 (7.7)/1/39 (2.6)/4/39 (10.3)	Patient satisfaction at 2 months (happy/OK/unsatisfactory/missing) Proportion responding ‘happy’ vs. all other responses *p* = 0.78
Clean‐contaminated surgery			
Khlifi et al. [25]	189/200 (94.5%)	140/200 (70%)	Satisfaction with medical care (‘yes’), *p* < 0.001
El‐Sayed et al. [27]	57/64 (89.1)/58/64 (90.6)/56/64 (87.5)/40/64 (62.3)	29/64 (45.3)/40/64 (62.5)/38/64 (59.4)/ 28/64 (43.8)	Sit easily (*p* < 0.001)/stand easily (*p* < 0.001)/walk easily (*p* < 0.001)/squatting (*p* = 0.034)
Tan et al. [22]	7 (5, 8) *n* = 165	7 (5, 8) *n* = 166	Satisfaction with wound management at hospital discharge, median (IQR), *p* = 0.81^a^
	8 (7, 8) *n* = 165	8 (7, 8) *n* = 166	Satisfaction with wound management on Day 14, median (IQR)^a^, *p* = 0.76
	9 (9, 10) *n* = 153	9 (9, 9) *n* = 157	Satisfaction with wound management on day 28, median (IQR)^a^, *p* = 0.45
Chandrasiri and Fernandopullae [28]	Mean (SD)^b^ 8.5 (0.8)/7.7 (0.6)/7.6 (0.6)/5.8 (0.7)	Mean (SD)^b^ 7.4 (0.6)/7.0 (0.6)/7.1 (0.8)/5.1 (0.5)	Sit‐up easily (*p* < 0.001)/get off bed easily (*p* < 0.001)/walk easily (*p* < 0.001)/squat easily (*p* < 0.001)
Peleg et al. [24]	121/160 (75.6)/30/160 (18.8)/9/160 (5.6)	91/160 (56.9)/47/160 (29.4)/22/160 (13.8)	Satisfaction with timing of dressing removal and ability to wash/shower thereafter (pleased and satisfied [OR 2.35 95% CI: 1.46, 3.79]/no difference [OR 0.73 95% CI: 0.54, 0.97]/displeased and unsatisfied [OR 0.56 95% CI: 0.32, 0.97])

^a^ 11‐point rating scale: 0 (very dissatisfied) to 10 (very satisfied).

^b^ Assessed by visual analogue scale 1–10.

#### 3.8.1 Meta‐Analysis: Patient‐Reported Outcomes

**FIGURE 6:**
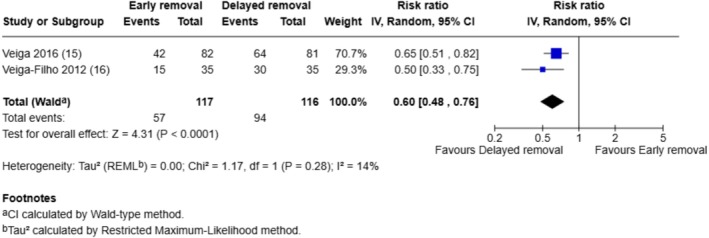
Meta‐analysis of responses rated as ‘excellent’ in terms of safety of dressing wear time.

**FIGURE 7:**
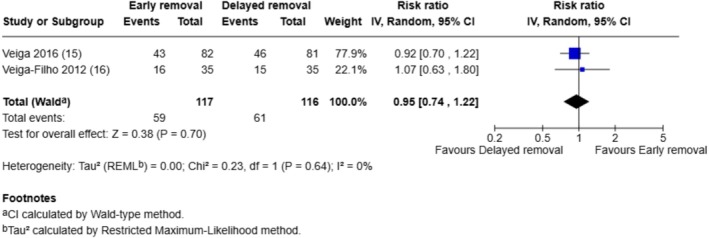
Meta‐analysis of responses rated as ‘excellent’ in terms of comfort of dressing wear time.

**FIGURE 8:**
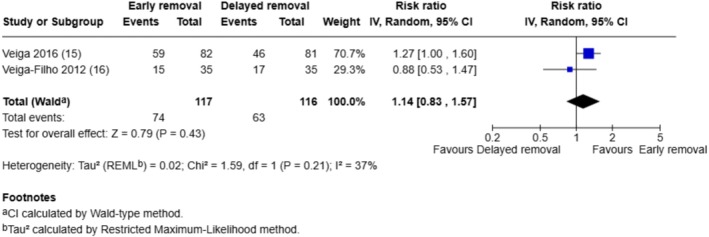
Meta‐analysis of responses rated as ‘excellent’ in terms of convenience of dressing wear time.

Two breast surgery trials that assessed patient‐reported perceptions of safety, comfort and convenience related to dressing wear time (positive outcomes) and we combined the responses rated as ‘excellent’ in a meta‐analysis (see Figures 6, 7, 8).

Significantly more participants whose dressings were removed after 6 days rated the safety of dressing wear time as ‘excellent’ compared to those whose dressings were removed after 1 day (RR 0.60, 95% CI: 0.48–0.76, *p* < 0.0001). There were no significant differences between the early and the delayed dressing removal groups in ratings of ‘excellent’ for perceptions of comfort (RR 0.95, 95% CI: 0.74–1.22, *p* = 0.70) or convenience related to dressing wear time (RR 1.14, 95% CI: 0.83–1.57, *p* = 0.43).

### 3.9 Ongoing Trials

Three relevant ongoing trials were identified. These trials vary in terms of sample size, type of surgery, duration of dressing and secondary outcome measures. A summary of their characteristics is presented in Table 5.

**TABLE 5:** Characteristics of ongoing trials.

Study ID/country	Participants/target sample size	Intervention vs. comparator	Outcomes	Est study start/est study completion
CTRI/2024/07/071751 [38]/ India	Clean and clean contaminated surgery (general surgery/obstetrics and gynaecology)/ 206	Early dressing removal (24–48 h) vs Delayed dressing removal (48 h)	Primary: Superficial and deep SSI at 30 days Secondary: Pain score, duration of hospital stay, wound healing assessment	30‐08‐2024/ NR
NCT06569862 [39]/France	Elective abdominal surgery/ 1288	Dressing removal at 1 day vs. Dressing removal at 6 days +/− 1 day	Primary: Superficial SSI at 30 days Secondary: Costs, repeat surgery, unscheduled nursing care, % patients asking for scars to be cover, length of hospital stay, quality of life (EQ‐5D‐5L)	10–2024/ 12–2026
NCT06263205 [40]/China	Gastrointestinal tumours/ 212	Dressing removal at 48 h vs Dressing removal at 7–14 days	Primary: Wound healing complications at 30 days (including SSI, fat liquefaction, wound dehiscence) Secondary: Pain, costs	01‐04‐2024/ 24‐12‐2026

## 4 Discussion

This systematic review assessed evidence from 16 RCTs comparing different lengths of time wounds are covered after surgery. The included studies encompassed a diverse range of surgical procedures, patient populations and dressing duration. All the included studies used standard dressings, had a relatively small sample size and reported low rates of SSI. It is worth noting that all included studies were assessed as being at either ‘high’ overall risk of bias for the outcome surgical site infection (SSI) or having ‘some concerns’ primarily due to the way SSI was measured or the absence of a prespecified statistical analysis plan. Similarly, the certainty of evidence for SSI was very low, with downgrading driven by heterogeneity in the overall effect estimate and wide confidence intervals in the individual studies.

The primary research question addressed whether the timing of dressing removal affects the incidence of SSI. When all 16 studies were combined in a meta‐analysis, there was no statistically significant difference in SSI rates between the early and delayed removal groups. The overall effect suggested a modest, non‐significant trend towards favouring early removal. However, the included studies were heterogeneous in terms of patient population, type of surgery and dressing duration. The analysis that focused on three trials involving breast surgery showed an opposite effect, with fewer SSI in the delayed removal groups, but the result also failed to reach statistical significance. This may indicate that the timing of dressing removal is context specific, with different anatomical regions requiring a different approach.

Length of hospital stay and length of time for wound healing tended to be similar for both early and delayed dressing removal groups, although some studies at high risk of bias showed shorter hospital stay and shorter wound healing time for the early removal groups. However, patient‐reported outcomes reported by a subset of breast surgery studies showed that perceptions of safety were more favourable with longer dressing durations. In these studies, comfort and convenience ratings did not differ meaningfully between groups. Patient satisfaction was similar between the early and delayed removal groups in two studies but significantly higher for the early removal group in a third study. Rates of wound dehiscence and unintended readmission to hospital were low across studies reporting those outcomes.

The secondary question—whether the optimal duration of wound coverage varies by dressing type, surgical procedure or wound characteristics—could not be answered due to insufficient data. Most trials did not provide subgroup analyses based on wound type, anatomical site or type of dressing material, limiting our ability to draw conclusions about context‐specific practices.

Our findings are generally in agreement with previous systematic reviews, which similarly concluded that early and delayed dressing removal are comparable in terms of SSI risk. Furthermore, earlier systematic reviews (i.e., Zhang et al. [1], Toon et al. [2]) showed a slight advantage for early dressing removal [1, 2]. However, our analyses by type of surgery revealed conflicting findings. Two breast surgery trials favoured delayed removal, [15, 16] while the eight caesarean section trials produced mixed results (see Figure 3) [21, 22, 23, 24, 25, 26, 27, 28]. Such variation may be attributed to the heterogeneity in trial populations (two trials recruited only ‘low risk’ participants), [21, 24] definition of SSI, duration of dressing and time of SSI assessment. None of the eight caesarean section trials were conducted in the UK, where the current clinical recommendation (NICE NG192) is the removal of dressing 6 to 24 h after caesarean birth [30]. These inconsistencies among existing trials challenge the notion of universal guidelines for wound dressing and highlight the need for tailored recommendations based on the type of surgery and clinical context. Indeed, published recommendations do exist in some countries (e.g., Australia, New Zealand and Japan) [31, 32, 33] for wound dressings after specific types of surgery, for example, head and neck surgery, [34] total joint arthroplasty [35] and lumbar fusion surgery [36].

### 4.1 Limitations of the Evidence Included in the Review

This review is limited by the methodological quality and heterogeneity of the included studies. Certainty of evidence relating to SSI was very low. Differences in study design, duration of intervention, outcome definitions and surgical procedures hampered the comparability of trials and the robustness of pooled estimates. Specifically, we were unable to draw any conclusions about the optimal duration of wound dressing due to the lack of consistency in the duration of dressing across the intervention and comparator groups. Also, the predominance of small trials and ‘high’ or ‘some concerns’ risk of bias further weakens our confidence in the observed findings. In addition, three studies [14, 15, 16] involved the same research team and investigated the same dressing duration (6 days vs. 1 day), potentially introducing duplication bias. However, the study periods do not appear to overlap.

### 4.2 Limitations of the Review Processes

A limitation at the review level is that, due to resource constraints, two reviewers did not operate fully independently throughout all stages of the review process. However, we employed consistent and reproducible methods that were prespecified in a research protocol. This review was originally intended to be a scoping review and was registered as such on Research Registry. Following consultation with stakeholders and clinical experts, the inclusion criteria were deemed sufficiently well‐defined to support a systematic review with formal risk of bias assessment. Although we do not consider this change in approach to be a methodological limitation, we acknowledge that, despite multiple attempts, we were unable to formally amend the registered protocol.

### 4.3 Implications for Practice, Policy and Future Research

At present, evidence is insufficient to support definite recommendations regarding the optimal duration of dressings to prevent SSI after surgery. Until stronger evidence emerges, practitioners and policymakers are advised to follow established guidelines (e.g., NICE NG125 and NG192) [30, 37] when determining dressing duration.

Future research should prioritise well‐powered, high‐quality randomised controlled trials (RCTs) that examine clearly defined and standardised dressing durations, with stratification by surgery type, wound characteristics and dressing material. These trials should also use consistent definitions of SSIs and standardised follow‐up periods to enable meaningful comparisons. In particular, adopting harmonised duration protocols across clinical trials would yield more reliable and generalisable findings. There is a pressing need for consensus among clinicians on which dressing durations warrant further investigation. Importantly, future studies should incorporate patient‐centred outcomes and measures of quality of life, as these play a vital role in postoperative care and patient adherence.

## Funding

This work was supported by the National Institute for Health and Care Research (NIHR167859).

## Ethics Statement

The authors have nothing to report.

## Conflicts of Interest

The authors declare no conflicts of interest.

## Supporting information


**Data S1:** iwj70943‐sup‐0001‐Supinfo.docx.

## Data Availability

The data that support the findings of this study are available from the corresponding author upon reasonable request.

## Appendix 1 Search Strategies

### Ovid MEDLINE(R) ALL <1946 to August 29, 2024>

1. bandages/ or occlusive dressings/
2. (dressing? or (cover* adj5 (wound? or incision? or dressing?))).tw,kf.
3. 1 or 2
4. Surgical Wound/
5. ((surg* or operative) adj5 (site? or wound? or incision?)).tw,kf.
6. Caesarean Section/ or c?esarean.tw.
7. 4 or 5 or 6
8. time factors/ or time.kf.
9. ((early or delay* or time? or timing or hour? or ‘h’ or day? or ‘d’ or beyond or after or within or duration or length) adj10 (dressing? or wound?)).tw.
10. 8 or 9
11. surgical wound infections/ or wound healing/ or surgical wound dehiscence/
12. Postoperative Complications/
13. bandages/ae or occlusive dressings/ae
14. Surgical Wound/co
15. ((surg* or operat*) adj5 (infect* or dehisc*)).tw,kf.
16. (wound? adj5 (infect* or dehisc* or fail*)).tw,kf.
17. ((post‐operative* or postoperative*) adj5 infect*).tw,kf.
18. Patient Readmission/ or readmission?.tw,kf.
19. ‘Length of Stay’/ or (length adj3 stay).tw,kf.
20. ‘Quality of Life’/ or (‘quality of life’ or QOL or HrQOL).tw,kf.
21. mortality/ or mortality.tw,kf
22. or/11‐21
23. 3 and 7 and 10 and 22
24. limit 23 to yr = ‘1989–Current’

### Ovid Embase <1974 to 2024 Week 34>

1. exp. ‘bandages and dressings’/
2. (dressing? or (cover* adj5 (wound? or incision? or dressing?))).tw,kf.
3. 1 or 2
4. surgical wound/
5. ((surg* or operative) adj5 (site? or wound? or incision?)).tw,kf.
6. Cesarean Section/ or c?esarean.tw.
7. 4 or 5 or 6
8. time factors/ or time.kf.
9. ((early or delay* or time? or timing or hour? or ‘h’ or day? or ‘d’ or beyond or after or within or duration or length) adj10 (dressing? or wound?)).tw.
10. 8 or 9
11. surgical infection/ or wound healing/ or wound dehiscence/
12. postoperative complication/
13. surgical wound/co, si [Complication, Side Effect]
14. ((surg* or operat*) adj5 (infect* or dehisc*)).tw,kf.
15. (wound? adj5 (infect* or dehisc* or fail*)).tw,kf.
16. ((post‐operative* or postoperative*) adj5 infect*).tw,kf.
17. hospital readmission/ or readmission?.tw,kf.
18. ‘Length of Stay’/ or (length adj3 stay).tw,kf.
19. ‘Quality of Life’/ or (‘quality of life’ or QOL or HrQOL).tw,kf.
20. mortality/ or mortality.tw,kf
21. or/11–20
22. 3 and 7 and 10 and 21
23. limit 22 to yr = ‘1989 –Current’
24. conference abstract.pt.
25. 23 not 24

### EBSCO CINAHL

S1 (MH ‘Bandages and Dressings+’)

S2 TX (dressing? OR (cover* N5 (wound? OR incision? OR dressing?)))

S3 S1 OR S2

S4 (MH ‘Surgical Wound’)

S5 TX ((surg* OR operative) N5 (site? OR wound? OR incision?))

S6 S4 OR S5

S7 (MH ‘Time Factors’)

S8 TX ((early OR delay* OR time? OR timing OR hour? OR ‘h’ OR day? OR ‘d’ OR beyond OR after OR within OR duration OR length) N10 (dressing? OR wound?))

S9 S7 OR S8

S10 (MH ‘Surgical Wound Dehiscence’) OR (MH ‘Surgical Wound Infection’) OR (MH ‘Wound Healing’)

S11 (MH ‘Postoperative Complications’)

S12 (MH ‘Bandages and Dressings+/AE’)

S13 (MH ‘Surgical Wound/CO’)

S14 TX ((surg* OR operat*) N5 (infect* OR dehisc*))

S15 TX (wound? N5 (infect* OR dehisc* OR fail*))

S16 TX ((post‐operative* OR postoperative*) N5 infect*)

S17 (MH ‘Readmission’) OR TX readmission?

S18 (MH ‘Length of Stay’) OR TX length N3 stay

S19 (MH ‘Quality of Life’) OR (TX (‘quality of life’ OR QOL OR HrQOL))

S20 (MH ‘Mortality’) OR TX mortality

S21 S10 OR S11 OR S12 OR S13 OR S14 OR S15 OR S16 OR S17 OR S18 OR S19 OR S20

S22 S3 AND S6 AND S9 AND S21

### Cochrane Library

#1 MeSH descriptor: [Bandages] this term only

#2 MeSH descriptor: [Occlusive Dressings] this term only

#3 (dressing? or (cover* NEAR/5 (wound? or incision? or dressing?))):ti,ab,kw

#4 #1 or #2 or #3

#5 MeSH descriptor: [Surgical Wound] this term only

#6 ((surg* or operative) NEAR/5 (site? or wound? or incision?)):ti,ab,kw

#7 #5 or #6

#8 MeSH descriptor: [Time Factors] this term only

#9 ((early or delay* or time? or timing or hour? or ‘h’ or day? or ‘d’ or beyond or after or within or duration or length) NEAR/10 (dressing? or wound?)):ti,ab,kw

#10 #8 or #9

#11 MeSH descriptor: [Surgical Wound Infection] this term only

#12 MeSH descriptor: [Wound Healing] this term only

#13 MeSH descriptor: [Surgical Wound Dehiscence] this term only

#14 MeSH descriptor: [Postoperative Complications] this term only

#15 MeSH descriptor: [Bandages] this term only and with qualifier(s): [adverse effects—AE]

#16 MeSH descriptor: [Occlusive Dressings] this term only and with qualifier(s): [adverse effects—AE]

#17 MeSH descriptor: [Surgical Wound] this term only and with qualifier(s): [complications—CO]

#18 ((surg* or operat*) NEAR/5 (infect* or dehisc*)):ti,ab,kw

#19 (wound? NEAR/5 (infect* or dehisc* or fail*)):ti,ab,kw

#20 ((post‐operative* or postoperative*) NEAR/5 infect*):ti,ab,kw

#21 MeSH descriptor: [Patient Readmission] this term only

#22 (readmission?):ti,ab,kw

#23 MeSH descriptor: [Length of Stay] this term only

#24 (length NEAR/3 stay):ti,ab,kw

#25 MeSH descriptor: [Quality of Life] this term only

#26 (‘quality of life’ or QOL or HrQOL):ti,ab,kw

#27 MeSH descriptor: [Mortality] this term only

#28 (mortality):ti,ab,kw

#29 #11 or #12 or #13 or #14 or #15 or #16 or #17 or #18 or #19 or #20 or #21 or #22 or #23 or #24 or #25 or #26 or #27 or #28

#30 #4 and #7 and #10 and #29

## Appendix 2 Characteristics of Included Studies

**Table:**

Study details	Inclusion criteria	Exclusion criteria
Clean surgery		
First author, year: Mendes 2018 [14] Secondary reports: Mendes 2015 [41] Country: Brazil Language: English Publication type: Full text Setting: Hospital Study design: RCT Intervention/duration: 4 layers of sterile cotton gauze covered with micropore tape/1 day Comparator/duration: 4 layers of sterile cotton gauze covered with micropore tape/6 days	Women aged between 18 and 60 years who are candidates for breast augmentation with silicone implants via the inframammary fold approach	Patients with a body mass index (BMI) above 30 kg/m^2^, those on immunosuppressive therapy, those with diabetes, those who smoke and those with a history of breast cancer or breast surgery were excluded. Patients with an indication for an areolar or axillar approach and those who had the dressing wet were also excluded
First author, year: Veiga 2016 [15] Secondary reports: No Country: Brazil Language: English Publication type: Full text Setting: Hospital Study design: RCT Intervention/duration: 4 layers of sterile cotton gauze covered with micropore tape/1 day Comparator/duration: 4 layers of sterile cotton gauze covered with micropore tape/6 days	Breast cancer patients aged 18 years and older who were undergoing immediate or delayed breast reconstruction by any technique	Patients who smoked heavily, had a body mass index (BMI) above 35 kg/m^2^, were diabetic, who had any contraindication for breast reconstruction procedures were excluded, as were patients whose dressing had gotten wet in the first 24 h after the operation
First author, year: Ghandi 2012 [17] Secondary reports: No Country: Iran Language: English Publication type: Full text Setting: Hospital Study design: RCT Intervention/duration: Vaselinated gauze and dry gauze/24 h Comparator/duration: Vaselinated gauze and dry gauze/48 h	Patients with facial melanocytic nevi, were candidates or volunteers for nevus excision, gave consent for participation in the study and their follow‐up was possible after surgery for evaluating wound infection	Patients with recent burn in the lesion sites, very obese patients (BMI > 35), patients receiving antibiotics for any reason, patients under treatment with systemic antibiotics after surgery, diabetic patients; patients with history of pulmonary disease, patients who had taken immunosuppressive drugs within the recent 3 months, patients under phototherapy in the recent 3 months; patients who needed flap for lesion reconstruction and patients who needed suturing of the subcutaneous tissue at the time of nevi excision
First author, year: Ritting 2012 [18] Secondary reports: No Country: USA Language: English Publication type: Full text Setting: Hospital Study design: RCT Intervention/duration: Medicated gauze and cotton gauze plus cast padding and elastic roller bandage/48–72 h Comparator/duration: Medicated gauze and cotton gauze plus cast padding and elastic roller bandage/2 weeks	Patients undergoing carpal tunnel release	NR
First author, year: Veiga‐Filho 2012 [16] Secondary reports: No Country: Brazil Language: English Publication type: Full text Setting: Hospital Study design: RCT Intervention/duration: 4 layers of sterile cotton gauze covered with micropore tape/1 day Comparator/duration: 4 layers of sterile cotton gauze covered with micropore tape/6 days	Patients over 18 years of age, scheduled for bilateral breast reduction for macromastia	Patients with a body mass index above 30 kg/m^2^, those with a breast cancer history, and those with the usual contraindications for elective plastic surgery procedures, such as diabetes or immunosuppression, were excluded, as well as heavy smokers (≥ 15 g of tobacco per day) and patients who did not stop smoking at least 4 weeks before surgery. Patients whose dressings got wet in the first 24 h after operation, requiring changing, were also excluded
First author, year: Heal 2006 [20] Secondary reports: No Country: Australia Language: English Publication type: Full text Setting: Primary care Study design: RCT Intervention/duration: Melolin dressing and tape/Within 12 h Comparator/duration: Melolin dressing and tape/48 h	All patients who presented to a participating general practitioner for ‘minor skin excision’, except for skin excisions on the face	Patients who were already taking oral antibiotics, for whom oral or topical antibiotics were clinically indicated immediately postoperatively, or who were on immunosuppressive drugs. Further exclusion criteria were lacerations, having a flap or two‐layer procedure (tying a ‘bleeder’ did not count as two layer), excision of a sebaceous cyst and skin excision on the face
First author, year: Ramkumar 2006 [19] Secondary reports: No Country: UK Language: English Publication type: Full text Setting: Hospital Study design: RCT Intervention/duration: Standard head bandage/12 h Comparator/duration: Standard head bandage/10 days	Children below 16 years of age, undergoing a unilateral or bilateral primary correction of prominent ears	Patients requiring secondary revision; those aged 16 years or above at the time of surgery and those with any other type of congenital ear deformity other than prominent ears
Clean‐contaminated surgery		
First author, year: Khlifi 2022 [25] Secondary reports: No Country: Tunisia Language: English Publication type: Full text Setting: One University hospital Study design: RCT Intervention/duration: Operative wounds covered by compressive dressing without local antiseptic/2nd post‐operative day Comparator/duration: Operative wounds covered by compressive dressing without local antiseptic, changed every 2 days/10th post‐operative day	≥ 28 weeks gestation, elective caesarean, out of work, intact amniotic sac and informed consent	Parturient in labour, Premature Rupture of Membranes (PRM), chorioamnionitis, documented infection in progress or within 15 days prior to caesarean section, infection with Human Immunodeficiency Virus (HIV), longterm corticotherapy, antibiotherapy in progress or in the previous 15 days of caesarean section and chronic diabetes. Parturients who had withdrawn their consent were excluded, as well as those who had not complied with the protocol, or who required post‐operative antibiotherapy because of concomitant infection (independent of the surgical site)
First author, year: Kilic 2021 [21] Secondary reports: No Country: USA and Turkey Language: English Publication type: Full text Setting: Three hospitals Study design: RCT Intervention/duration: Incisions covered with telfa. Gauze pads placed over the telfa and covered with Medipore tape/24 h Comparator/duration: Incisions covered with telfa. Gauze pads placed over the telfa and covered with Medipore tape/48 h	Low‐risk obstetric patients aged 18–44 years with term, singleton pregnancies who planned to have Caesarean Delivery (CD). CD indications, such as scheduled non‐laboured primary CD for foetal malpresentation, suspected macrosomia, maternal request placental anomaly and abnormal/indeterminate foetal heart tracing without labour, were included. In addition, first, second and third repeat CDs were included	Preeclampsia, preeclampsia with severe features, eclampsia, known preoperative infectious disease, any unknown origin preoperative fever, diabetes, pregnant with premature rupture of membrane (PROM) or rupture of membrane (ROM), intraoperative findings suggestive of an underlying cancerous condition, vertical skin incision planned hysterectomy during CD
First author, year: Wadhwa 2021 [26] Secondary reports: No Country: India Language: English Publication type: Full text Setting: Tertiary care centre Study design: RCT Intervention/duration: Wound covered with 2 pieces of sterile gauze and waterproof adhesive bandage or plaster/4 days Comparator/duration: Wound covered with 2 pieces of sterile gauze and waterproof adhesive bandage or plaster/8 days	Antenatal women aged between 18 to 40 years who underwent caesarean section	The cases with obstructed labour, chorioamnionitis, intrauterine death and fever were excluded from study
First author, year: El‐Sayed 2020 [27] Secondary reports: No Country: Egypt Language: English Publication type: Full text Setting: Private hospital Study design: RCT Intervention/duration: Standard gauze dressing covered with Elastoplast/6–12 h Comparator/duration: Standard gauze dressing covered with Elastoplast/5 days	Women undergoing their first caesarean section; not had complications during pregnancy	Previous surgical site skin infection (SSI); pyrexia before surgical procedure; body mass index (BMI) of 35 kg/m^2^ or more; history of Elastoplast hypersensitivity
First author, year: Tan 2020 [22] Secondary reports: No Country: Malaysia Language: English Publication type: Full text Setting: Hospital Study design: RCT Intervention/duration: No dressing/no duration Comparator/duration: Standard low adhesive film with absorbent pad dressing/1 day	Participants aged ≥ 18 years scheduled for caesarean section (planned or unplanned) with a prenatal test negative for HIV and Hepatitis B	Any other known blood or bodily secretion transmissible infection, category 1 (very urgent) caesarean section, midline incision and no phone access. After the closure of the skin layer, the surgeon‐in‐charge decided whether coverage (e.g., with haemostatic pressure dressing or support dressing) of the wound was clinically indicated—this represented the final exclusion criterion
First author, year: Nesrallah 2017 [23] Secondary reports: No Country: USA Language: English Publication type: Conference abstract Setting: Hospital Study design: RCT Intervention/duration: NR/12–30 h Comparator/duration: NR/30–48 h	Patients with viable pregnancies at 23 0/7 weeks of gestation or greater undergoing scheduled and unscheduled caesarean delivery	NR
First author, year: Chandrasiri 2016 [28] Secondary reports: No Country: Sri Lanka Language: English Publication type: Full text Setting: One hospital Study design: RCT Intervention/duration: Standard gauze dressing covered with Elastoplast/6–12 h Comparator/duration: Standard gauze dressing covered with Elastoplast/24–30 h	Patients undergoing caesarean section	Prior surgical site skin infection, pyrexia before surgery, BM I ≥ 35, history of Elastoplast allergy, skin incision other than a Joel Cohen incision and caesarean resulting in additional procedures due to complications
First author, year: Peleg 2016 [24] Secondary reports: No Country: Israel Language: English Publication type: Full text Setting: Hospital Study design: RCT Intervention/duration: Standard non‐woven wound dressing/6 h Comparator/duration: Standard non‐woven wound dressing/24 h	Low‐risk women aged 18–44 years at term with singleton pregnancies having a scheduled non‐laboured primary, first repeat or second repeat caesarean delivery and failed induction	Women with known pregnancy complications such as fever, diabetes or preeclampsia and patients who had laboured or with prelabour rupture of membranes. Also excluded were women with a body mass index ≥ 35.
First author, year: Zhou 2012 [29] Secondary reports: No Country: China Language: English Publication type: Full text Setting: Hospital Study design: RCT Intervention/duration: Dry sterile cotton gauze/48 h Comparator/duration: Dry sterile cotton gauze/7–8 days	Patients undergoing elective thoracic surgery	Unable to provide written consent, immunosuppressed or under the care of surgeons who did not wish to have their patients participate in this study. Patients receiving steroids or chemotherapy before or immediately after the surgery were also excluded.

## Appendix 3 Baseline Characteristics of Participants in Included Studies

**Table:**

Study ID (country)	Group (*n* randomised /n analysed)	Age, years, mean (SD)	Sex, *n* (%)	Type of surgery, *n* (%)	BMI, kg/m^2^, mean (SD)	Antibiotics administered, *n* (%)
Clean surgery						
Mendes et al. [14] (Brazil)	1 day (40/40)	27.9 (9.2)	F, 40 (100)	Breast augmentation, 40 (100)	21.8 (2.5)	Prophylactic antibiotics, 40 (100) Before start of anaesthesia and every 6 h during hospitalisation
	6 days (40/38)	30.9 (10.0)	F, 40 (100)	Breast augmentation, 40 (100)	22.4 (2.2)	Prophylactic antibiotics, 40 (100) Before start of anaesthesia and every 6 h during hospitalisation
Veiga et al. [15] (Brazil)	1 day (100/94)	47.8 (9.5)	F, 100 (100)	Breast reconstruction immediate, 58 (58) or delayed, 42 (42)	25.8 (2.9)	Prophylactic antibiotics, 100 (100) At induction of anaesthesia and every 6 h postoperatively for first 24 h
	6 days (100/92)	49.3 (9.3)	F, 100 (100)	Breast reconstruction immediate, 48 (48) or delayed, 52 (52)	25.2 (2.9)	Prophylactic antibiotics, 100 (100) At induction of anaesthesia and every 6 h postoperatively for first 24 h
Ghandi et al. [17] (Iran)	24 h (234 nevi/234 nevi)	31 (6.9)	Total *n* = 222* F, 172 (77.5) M, 50 (22.5)	Facial melanocytic excision, 222 (100)	NR	0 (0)
	48 h (234 nevi/234 nevi)	28 (8.1)			NR	0 (0)
Ritting et al. [18] (USA)	48–72 h (45/45 at visit 1; 30 analysed at visit 2)	46.3 (14.8)	F, 31 (69) M, 14 (31)	Carpal tunnel release, 45 (100)	33.1 (8)	NR
	2 weeks (49/49 at visit 1; 36 analysed at visit 2)	44.8 (12.3)	F, 42 (86) M, 7 (14)	Carpal tunnel release, 49 (100)	33.9 (8.5)	NR
Veiga‐Filho et al. [16] (Brazil)	1 day (35/35)	Median (range) 34 (18, 54)	F, 35 (100)	Bilateral breast reduction, 35 (100)	Median (range) 25.3 (22.4, 29.5)	Prophylactic antibiotics, 0 (0); Antibiotics for SSI, 7 (20)
	6 days (35/35)	Median (range) 34 (18, 59)	F, 35 (100)	Bilateral breast reduction, 35 (100)	Median (range) 25.4 (20.8, 29.3)	Prophylactic antibiotics, 0 (0); Antibiotics for SSI, 2 (5.7)
Heal et al. [20] (Australia)	12 h (450/450)	55.9 (16.6)	F, 201 (44.7) M, 249 (55.3)	Minor skin excision, 450 (100); including excision of skin cancer, 294 (65), excision at lower limb, 112 (25)	NR	0 (0)
	48 h (420/420)	56.5 (16.2)	F, 212 (50.5) M, 208 (49.5)	Minor skin excision, 420 (100); including excision of skin cancer, 289 (69), excision at lower limb, 106 (25)	NR	0 (0)
Ramkumar et al. [19] (UK)	24 h (39/35)	10 (3)	F, 17 (44) M, 22 (56)	Pinnaplasty, 39 (100)	NR	NR
	10 days (39/32)	9 (3)	F, 21 (54) M, 18 (46)	Pinnaplasty, 39 (100)	NR	NR
Clean‐contaminated surgery						
Khlifi et al. [25] (Tunisia)	2 days (NR/200)	32.5 (5.2)	F, 200 (100)	CS, 200 (100)	≤ 25: 6% 25–29: 29% ≥ 30: 65%	Prophylactic antibiotics, 200 (100) Single IV dose 30 min before incision
	10 days (NR/200)	32.3 (5.2)	F, 200 (100)	CS, 200 (100)	≤ 25: 10% 25–29: 31% ≥ 30: 65%	Single IV dose 30 min before incision
Kilic et al. [21] (USA and Turkey)	24 h (531/430)	29.2 (5.8)	F, 531 (100)	CS, 531 (100)	29.7 (4.2)	Prophylactic antibiotics, 531 (100) Single IV dose within 60 min before incision
	48 h (526/439)	29.4 (5.5)	F, 526 (100)	CS, 526 (100)	30.0 (4.9)	Prophylactic antibiotics, 526 (100) Single IV dose within 60 min before incision
Wadhwa et al. [26] (India)	4 days (250/250)	29.8 (4.7)	F, 250 (100)	CS, 250 (100)	22.8 (2.3)	Per‐operative antibiotics for at least 5d
	8 days (250/250)	28.1 (4.5)	F, 250 (100)	CS, 250 (100)	23.4 (2.2)	Per‐operative antibiotics for at least 5d
El‐Sayed et al. [27] (Egypt)	6–12 h (64/64)	18–23, 37.5% 24–29, 54.7% 30–35, 7.8%	F, 64 (100)	CS, 64 (100)	NR	NR
	5 days (64/64)	18–23, 32.8% 24–29, 64.1% 30–35, 3.1%	F, 64 (100)	CS, 64 (100)	NR	NR
Tan et al. [22] (Malaysia)	Exposed^**^ (167/153)	31.8 (4.4)	F, 167 (100)	CS, 165 (100)	30.1 (5.4)	Prophylactic antibiotics, 165 (100) 3 doses, 1st just before incision
	1 day^**^ (167/157)	31.8 (4.4)	F, 167 (100)	CS, 166 (100)	29.4 (5.7)	Prophylactic antibiotics, 166 (100) 3 doses, 1st just before incision
Nesrallah et al. [23] (USA)	12–30 h (300/NR)	NR	NR	CS, NR (100)	NR	NR
	30–48 h (302/NR)	NR	NR	CS, NR (100)	NR	NR
Chandrasiri and Fernandopullae [28] (Sri Lanka)	6–12 h (205/205)	29.6 (4.8)	F, 205 (100)	CS, 205 (100)	24.6 (3.1)	Prophylactic antibiotics, 205 (100)
	24–36 h (195/195)	29.8 (4.9)	F, 195 (100)	CS, 195 (100)	24.2 (3.2)	Prophylactic antibiotics, 195 (100)
Peleg et al. [24] (Israel)	6 h (160/160)	32.9 (5.3)	F, 160 (100)	CS, 160 (100)	30.9 (6.2)	Prophylactic antibiotics, 160 (100) Within 1 h of incision
	24 h (160/160)	31.6 (4.7)	F, 160 (100)	CS, 160 (100)	29.8 (5.5)	Prophylactic antibiotics, 160 (100) Within 1 h of incision
Zhou et al. [29] (China)	48 h (115/115)	54.1 (10.1)	F, 49 (42.6) M, 66 (57.4)	Lung cancer, 33 (28.7) Tumour of mediastinum, 26 (22.6) Pulmonary cysts, 16 (13.9) Diaphragmatic hernia, 15 (13.0) Oesophageal cancer, 15 (13.0) Oesophageal diverticulum, 10 (8.7)	25.6 (3.7)	Prophylactic antibiotics, 115 (100) Cefazolin 1.0 administered intravenously every 8 h postoperatively for 48 h, or until all chest draining tubes were removed
	7–8 days (115/115)	52.4 (12.6)	F, 54 (47.0) M, 61 (53.0)	Lung cancer, 30 (26.1) Tumour of mediastinum, 25 (21.7) Pulmonary cysts, 18 (15.7) Diaphragmatic hernia, 12 (14.3) Oesophageal cancer, 17 (14.8) Oesophageal diverticulum, 13 (11.7)	26.2 (4.0)	Prophylactic antibiotics, 115 (100) Cefazolin 1.0 administered intravenously every 8 h postoperatively for 48 h, or until all chest draining tubes were removed

*Note:* *In terms of nevi, in 24 h group: Female 192/234 (82.1%); Male 42/234 (17.9%). In 48 h group: Female 183 (78.2%); Male 51/234 (21.8). **167 randomised in each group but 2 in the exposed group were dressed mistakenly and 1 in the dressed group was exposed mistakenly so 165 and 166, respectively, continued in the trial.
