# Supplementary material for: Duration of Wound Coverage for the Prevention of Surgical Site Infections After Surgery: A Systematic Review of Current Evidence With Meta‐Analysis of Randomised Controlled Trials
Source: Int Wound J. 2026 May 12;23(5):e70943. doi: 10.1111/iwj.70943 (PMC13167696; doi:10.1111/iwj.70943)
Supplement: Supplementary file 1 — Data S1: iwj70943‐sup‐0001‐Supinfo.docx. [file IWJ-23-e70943-s001.docx]

## SUPPLEMENT Risk of bias assessments of individual studies

| **Study ID** | **Experimental** | **Comparator** | **Randomisation process** | **Deviations from intended** | **Missing outcome data** | **Measurement of outcome** | **Selection of reported results** | **Overall** |
| --- | --- | --- | --- | --- | --- | --- | --- | --- |
| Mendes 2018 | 1d | 6d |  |  |  |  |  |  |
| Veiga 2016 | 1d | 6d |  |  |  |  |  |  |
| Ghandi 2012 | 24h | 48h |  |  |  |  |  |  |
| Ritting 2012 | 48-72h | 2w |  |  |  |  |  |  |
| Veiga-Filho 2012 | 1d | 6d |  |  |  |  |  |  |
| Heal 2006 | 12h | 48h |  |  |  |  |  |  |
| Ramkumar 2006 | 12h | 10d |  |  |  |  |  |  |
| Khlifi 2022 | 2d | 10d |  |  |  |  |  |  |
| Kilic 2021 | 24h | 48h |  |  |  |  |  |  |
| Wadhwa 2021 | 4d | 8d |  |  |  |  |  |  |
| El-Sayed 2020 | 6-12h | 5d |  |  |  |  |  |  |
| Tan 2020 | Exposed | 1d |  |  |  |  |  |  |
| Nesrallah 2017 | 12-30h | 30-48h |  |  |  |  |  |  |
| Chandrasiri 2016 | 6-12h | 24-36h |  |  |  |  |  |  |
| Peleg 2016 | 6h | 24h |  |  |  |  |  |  |
| Zhou 2012 | 48h | 7-8d |  |  |  |  |  |  |
